# A specific hybridisation internalisation probe (SHIP) enables precise live-cell and super-resolution imaging of internalized cargo

**DOI:** 10.1038/s41598-021-04544-6

**Published:** 2022-01-12

**Authors:** Sara Hernández-Pérez, Pieta K. Mattila

**Affiliations:** 1 Institute of Biomedicine and MediCity Research Laboratories, University of Turku, Turku, Finland; 2 Turku Bioscience, University of Turku and Åbo Akademi University, Turku, Finland; 3 InFLAMES Research Flagship Center, University of Turku, Turku, Finland

**Keywords:** Super-resolution microscopy, Adaptive immunity, Antigen processing and presentation, Membrane trafficking

## Abstract

Facilitated by the advancements in microscopy, our understanding of the complexity of intracellular vesicle traffic has dramatically increased in recent years. However, distinguishing between plasma membrane-bound or internalised ligands remains a major challenge for the studies of cargo sorting to endosomal compartments, especially in small and round cells such as lymphocytes. The specific hybridization internalisation probe (SHIP) assay, developed for flow cytometry studies, employs a ssDNA fluorescence internalisation probe and a complementary ssDNA quenching probe to unambiguously detect the internalized receptors/cargo. Here, we adopted the SHIP assay to study the trafficking of receptor/ligand complexes using B lymphocytes and B cell receptor-mediated antigen internalization as a model system. Our study demonstrates the potential of the SHIP assay for improving the imaging of internalized receptor/ligand complexes and establishes the compatibility of this assay with multiple imaging modalities, including live-cell imaging and super-resolution microscopy.

## Introduction

Intracellular membrane trafficking has not only critical housekeeping functions but also controls various cell-type-specific functions regulating, for instance, signalling cascades and cell fate. Over the years, a general view of the vesicle pathways has been relatively well established. In particular, in the last decade, improvements in fluorescence microscopy have aided the discovery of different vesicle subpopulations and transport machineries. These improvements have increased our understanding of the location and behaviour of different vesicle populations, not possible to decipher otherwise using biochemical techniques^1–4^*.* Nevertheless, despite the improvements, imaging of vesicular pathways is challenged by the dynamic and heterogeneous nature of the vesicles and vesicular networks in all cell types. A particular challenge is confronted in small and round cells, such as lymphocytes, where the reduced size of the cytoplasm and the resulting immediate vicinity of the plasma membrane generate major obstacles for imaging approaches.

B lymphocytes, or B cells, are a crucial part of the adaptive immune system, initiating the antibody responses against a vast repertoire of different antigens. Precise coordination of the endosomal machinery is essential for processing the recognised antigens and triggering antibody responses. Through their B cell receptor (BCR), B cells recognise specific antigens that are internalized and directed to a specialized vesicular pathway. This antigen processing pathway enables controlled digestion of antigens into peptides that are then loaded onto the major histocompatibility complex class II (MHCII)^5^. The peptide-MHCII (pMHCII) complex is transported back to the cell surface and recognized by cognate CD4^+^ lymphocytes (T helper cells, T_H_ cells), providing a second activation signal to B cells. Reciprocally, pMHCII recognition also stimulates the cognate T_H_ cells to orchestrate other branches of the immune system and generate CD4^+^ T cell memory^6^. Hence, antigen internalisation and processing is a critical process that involves specialized endosomal compartments, and it needs to be tightly regulated to trigger B cell differentiation and effector T cell responses.

In our previous work, we characterised the antigen processing route in B cells by analysing different vesicle markers using spinning disk confocal microscopy (SDCM)^7^. However, unambiguous distinction between membrane BCRs and newly internalised BCRs remains a major obstacle in imaging approaches, as BCR is the most abundant receptor on the B cell surface (100,000–300,000 copies per cell)^8,9^ and engaged BCRs often form clusters on the cell membrane. Inaccurate determination of the extracellular and intracellular signal can ultimately skew the analyses or misguide the interpretations of the data. Traditionally, methods such as stripping of membrane proteins or treatment with proteases (i.e. trypsin) have been used to address similar problems^10,11^. However, some proteins can resist the stripping procedure, and these methods can compromise cell viability. Anti-fluorochrome antibodies can also be used to quench non-internalised fluorochromes, but this approach is limited to a few fluorophores, such as Alexa Fluor 488, Alexa Fluor 594, or fluorescein isothiocyanate (FITC)^12,13^. Alternatively, the non-internalized pool can be labelled prior to permeabilization to distinguish it from the internalized pool (for example, Fig. S2A–C in Hernández-Pérez et al.^7^). Nevertheless, quantification of the internalised antigen using this approach is time-consuming and not straightforward, and notably, none of these methods is compatible with live imaging. In an effort to address this problem, an elegant approach based on photoactivation (PA) of fluorescent proteins was recently used to visualize and quantify endocytic trafficking of T-cell surface receptors^14^. As a downside, the expression of an exogeneous proteins as well as a suitable high-end microscope setup is required in this method.

In 2013, Liu and Johnston developed an elegant method termed specific hybridization internalization probe (SHIP) assay to differentiate between internalised and non-internalised material in live cells^15^. This assay utilises a short (20-mer) single-stranded DNA (ssDNA) fluorescent internalization probe (FIP) coupled to the ligand of interest and a complementary ssDNA quenching probe (QP). Therefore, the SHIP assay can be used to investigate the kinetics of protein internalisation, and it has been employed in the past to analyse the nanoparticle internalization, antibody-dependent phagocytosis, and MHCII turnover in dendritic cells^16–19^. Notably, the SHIP system has been used almost exclusively in high-throughput flow cytometry studies, leaving its potential for imaging unexplored.

Here, we labelled antibodies recognising the IgM BCR expressed in A20 B cells with an Abberior® STAR 635P- or ATTO 647N-conjugated FIP to test the applicability of the SHIP system to the study of antigen trafficking in B cells not only in conventional confocal imaging but also in live and super-resolution imaging. We report efficient abolishment of the membrane-bound signal by the quencher probe, clearly enhancing the detection of internalized antigen vesicles, and provide a general proof-of-concept of the power of the SHIP assay for imaging receptor-mediated internalization and vesicle traffic using different microscopy modalities, including live imaging and super-resolution microscopy.

## Materials and methods

### Cells and transfections

A20 mouse lymphoma cells stably expressing a hen egg lysozyme (HEL)–specific IgM BCR (D1.3)^20^ were maintained in complete RPMI (cRPMI; RPMI 1640 with 2.05 mM l-glutamine supplemented with 10% fetal calf serum (FCS), 50 μM β-mercaptoethanol, 4 mM l-glutamine, 10 mM HEPES and 100 U/ml Penicillin/Streptomycin).

A20 D1.3 cells were transfected as previously described^21^. Briefly, 4 × 10^6^ cells were resuspended in 180 µl of 2S transfection buffer (5 mM KCl, 15 mM MgCl_2_, 15 mM HEPES, 50 mM sodium succinate, 180 mM Na_2_HPO_4_/ NaH_2_PO_4_ pH 7.2) containing 4 µg of plasmid(s) and electroporated using the AMAXA electroporation machine (program X-005, Biosystem) in 0.2 cm gap electroporation cuvettes. Cells were then transferred to 4 ml of pre-warmed cRPMI to recover overnight. GFP-Rab5 and RFP-Rab7 plasmids were a kind gift from Prof. Johanna Ivaska (University of Turku, Finland).

### Preparation of the fluorescence internalisation probe (FIP)

The fluorescent FIP-azide probes (5′ Abberior® STAR 635P- or ATTO 647N-TCAGTTCAGGACCCTCGGCT-N3 3′) and the quenching probe (QP; 5′ AGCCGAGGGTCCTGAACTGA-BHQ3 3′) were purchased from BioSynthesis Inc. (Lewisville, TX, USA) and dissolved in nuclease-free water. The donkey anti-mouse IgM antibodies (Jackson Immunoresearch; 715-005-020) were functionalized with a 10-fold molar excess of Click-iT™ SDP Ester sDIBO alkyne (Thermo, C20025) for 2 h at RT. Antibodies were then purified using a 7K Zeba spin desalting column (Thermo Scientific, 89882). After desalting, the functionalised antibodies were incubated with a 2-fold molar excess of fluorescent-FIP-azide at 4 °C O/N and purified using a 50 K MWCO Amicon filter (Merk Millipore, UFC5050). As a control, the same donkey anti-mouse IgM antibody was labelled with a 3-fold molar excess of ATTO 647 NHS (Sigma-Aldrich, 07376-1MG-F) and dialysed O/N at 4 °C. Effective labelling of the Abberior STAR 635P FIP-labelled anti-IgM (Abberior-FIP-IgM), the ATTO 647N FIP-labelled anti-IgM (ATTO-FIP-IgM) and the ATTO 647 anti-IgM antibodies was confirmed by NanoDrop and flow cytometry.

### BCR signalling and western blot

A20 D1.3 cells (10^5^ cells/condition) were starved in 90 µl of plain RPMI for 20 min and then different stimuli were added (10 µl/sample in plain RPMI): donkey anti-mouse IgM (final concentration (f_c_) 10 µg/ml), ATTO-FIP-IgM (f_c_ 10 µg/ml), Abberior-FIP-IgM (f_c_ 10 µg/ml) or FIP (f_c_ 100 nM; 5′ TCAGTTCAGGACCCTCGGCT-N3 3′, Metabion Oligos). As a control, 10 µl of RPMI were added to one of the samples (non-activated control). After 15 min, activation was stopped on ice, and 4X Laemmli buffer with beta-mercaptoethanol was added to a final concentration of 1X. Lysates were sonicated and boiled at 96 °C for 5 min. Samples were run on a 10% PAGE-SDS gel (25 μl/well) and transferred to a PVDF membrane (Trans-Blot Turbo Transfer System, Bio-Rad). Membranes were blocked with 5% milk in TBST (TBS, pH ~ 7.4 with 0.05% Tween-20) for 1 h and incubated O/N with primary antibodies (1:1.000; Cell Signalling Technologies #2938S, #3571S, #2701P) in 5% BSA in TBST at 4 °C. Anti-rabbit HRP (1:20.000; Jackson Immunoresearch 111-035-144) was added for 1 h at RT in 5% milk in TBST. Five washing steps (5 min each) were done with 10 ml of TBST. Membranes were incubated for 5 min with Immobilon Western Chemiluminescent HRP Substrate (WBKLS0500, Millipore) and imaged using a ChemiDoc MP Imaging System (Bio-Rad).

### Flow Cytometry

#### Measurement of antigen internalisation by flow cytometry

A20 D1.3 cells (10^7^/ml) were stained for 5 min on ice with biotinylated anti-IgM (Southern Biotech, 1021-08), Alexa Fluor® 633 anti-IgM (Jackson Immunoresearch, 715-605-14), FITC anti-IgM (Jackson Immunoresearch, 715-095-140), ATTO 647 anti-IgM, Abberior-FIP-IgM or ATTO-FIP-IgM. Cells (5 × 10^4^/well, 96-wp) were then incubated at 37 °C and 5% CO_2_ for 45, 30, 15 and 5 min. As a control (time 0), samples were kept on ice at all times. After incubation, cells activated with biotinylated anti-IgM were stained with Alexa Fluor® 633 streptavidin (Life Technologies, S-21375) and cells activated with FIP-IgM were quenched using 40 µl of 10 µM QP in PBS on ice for 20 min. Samples were then washed with cold PBS and analysed. A BD LSR Fortessa analyser equipped with four lasers (405, 488, 561, and 640 nm) was used. Data were analysed using FlowJo v10 (Tree Star). The internalisation rate for the FIP-IgM samples was calculated as follows:

1 $$\% \,antigen\,on\,the\,cell\,surface = 100 - \left( {\frac{{F_{Q} - F_{bg} }}{{F_{noQ} - F_{bg} }}} \right) \times 100$$

where *F*_*Q*_ represents the signal after quenching and *F*_*noQ*_ the signal before quenching for every given time point, and *F*_*bg*_ the background signal. The internalisation rate for the biotinylated anti-IgM samples was calculated as follows:

2 $$\% \,antigen\,on\,the\,cell\,surface = \left( {\frac{{F_{x} - F_{bg} }}{{F_{0} - F_{bg} }}} \right) \times 100$$

where *F*_*x*_ represents the signal at time x, *F*_0_ the signal at time 0 (non-internalised control; 100%), and *F*_*bg*_ the background signal.

### Flow cytometry

#### Measurement of cell viability by flow cytometry

A20 D1.3 cells (10^7^/ml) were transferred to a 96-well plate and treated with PBS, QP in PBS, acid wash buffer (HCl pH 2.0, 0.03 M sucrose, 10% FBS in PBS) or control buffer (pH 7.0, 0.03 M sucrose, 10% FBS in PBS ) for 1 min. Cells were then centrifuged (1300 rpm, 1 min) and stained with eBioscience™ Fixable Viability Dye eFluor™ 780 (1:1000) in PBS for 15 min on ice. Samples were then washed with cold PBS and the percentage of viable cells was analysed. A BD LSR Fortessa analyser equipped with four lasers (405, 488, 561, and 640 nm) was used. Data were analysed using FlowJo v10 (Tree Star).

### Microscopy samples

#### Visualisation of antigen vesicles in fixed samples

A20 D1.3 cells (10^7^/ml) were surface-labelled with 10 µg/ml of Abberior-FIP-IgM or ATTO-FIP-IgM in Imaging Buffer (10% FCS in PBS) for 5 min on ice. When indicated, cells were labelled simultaneously with Alexa Fluor® 488 donkey anti-mouse IgM F(ab)’_2_ (Jackson Immunoresearch, 715–546-020). Cells were then washed with PBS to remove excess unbound antigen and resuspended in Imaging Buffer (10^6^/ml). **SDCM and Airyscan:** After washing, cells (2 × 10^4^/well) were activated for different time points in an incubator (5% CO_2_, 37ºC) in a 12-well fibronectin-coated (80 ng/well) PTFE diagnostic slide (Fisher Scientific, 10028210)^22^. After activation, internalisation was stopped by placing the samples on ice. Samples were incubated on ice for 5–10 min in Quenching solution (10 µl of 1 µM QP in Imaging Buffer), followed by fixation with 4% PFA for 10 min at RT. Samples were then blocked and permeabilised with blocking buffer (5% donkey serum [Jackson Immunoresearch, 017-000-121] and 0.3% Triton X-100 in PBS) for 20 min at RT. After blocking, samples were stained with goat anti-EEA1 (1:100; Santa Crux, sc-6415) and rabbit anti-Rab7 (Cell Signalling, #9367) antibodies for 1 h at RT or 4ºC O/N in staining buffer (1% BSA, 0.3% Triton X100 in PBS), followed by staining with the secondary antibodies (1:500; Alexa Fluor® 488 donkey anti-rabbit IgG and Alexa Fluor® 555 donkey anti-goat) in PBS for 30 min at RT. Samples were washed with PBS (× 3 times) and mounted using FluoroMount-G containing DAPI (Thermo, 00495952). **STED:** After washing, cells (2 × 10^5^/coverslip) were activated for different time points in an incubator (5% CO_2_, 37ºC) on fibronectin-coated high-precision coverslips (Marienfeld, 0117650). After activation, internalisation was stopped by placing the samples on ice. Samples were incubated on ice for 5–10 min in Quenching solution (100 µl of 1 µM QP in Imaging Buffer), followed by fixation with 4% PFA for 10 min at RT. Samples were washed once with PBS and mounted on a slide (Thermo Scientific, J1800AMNZ) using ProLong™ Diamond Antifade Mountant (Thermo Fisher, P36970). Samples were imaged after curing for at least 20 h at RT.

#### Visualisation of antigen vesicles in live samples

For live imaging, unlabelled cells, cells labelled with 100 nM LysoTracker™ Green DND-26 (Thermo, L7526; 10 min at 37 °C) or cells transfected with GFP-Rab5 and RFP-Rab7 were used. A20 D1.3 cells (10^6^/ml; 100 µl per well) were seeded on a fibronectin-coated (500 ng/well) MatTek dish (MatTek, P35G-1.5-10-C) for 30 min in an incubator (5% CO_2_, 37ºC). Dishes were washed with PBS to remove unbound cells and transferred to ice. Cells were then stained with 10 μg/ml ATTO-FIP-IgM in Imaging Buffer (10% FCS in PBS) for 5–10 min on ice. When indicated, cells were simultaneously labelled with Alexa Fluor® 488 donkey anti-mouse IgM F(ab)’_2_. Samples were washed and transferred to the environmental chamber of the microscope (37 ºC) and imaged. Quencher was added (10 µl of 10 µM QP in Imaging Buffer) at different time points.

### Image acquisition and processing

#### Spinning disk confocal microscopy

Images were acquired using a 3i (Intelligent Imaging Innovations) Marianas spinning disk confocal system built on an inverted Zeiss Cell Observed microscope equipped with a Yokogawa CSU-W1 confocal scanner unit. The microscope was controlled using the SlideBook software. For fixed samples, the following objective, camera, laser lines and filters were used: an oil-immersion 63 × Zeiss Plan-Apochromat (1.4 NA, working distance 0.19 mm) objective, a Photometrics Prime BSI back-illuminated scientific CMOS camera (2048 × 2048 pixels, 1 × 1 binning, pixel size 6.5 × 6.5 µm), 405 (100 mW), 488 (150 mW), 561 (100 mW) and 640 (100 mW) nm laser lines and 445/45 nm (DAPI), 525/30 nm (Alexa Fluor® 488), 617/73 nm (Alexa Fluor® 555) and 692/40 nm (Alexa Fluor® 647) filters. For live imaging, the following objective, camera, laser lines and filters were used: an oil-immersion 63x Zeiss Plan-Apochromat (1.4 NA, working distance 0.17 mm), a Photometrics Evolve Delta EMCCD (512 × 512 pixels, 1 × 1 binning, pixel size 16 × 16 µm) camera, 488 (150 mW), 561 (150 mW) and 640 (100 mW) nm laser lines and 525/30 nm (Alexa Fluor® 488), 617/73 nm (Alexa Fluor® 555) and 692/40 nm (Alexa Fluor® 647) filters. The gain was set to 1, and laser power to 30%. The microscope was equipped with an Okolab temperature control and environment chamber for live imaging.

#### Airyscan confocal microscopy

Images were acquired using a laser scanning confocal microscope LSM880 (Zeiss) with an Airyscan detector equipped with 405 (Diode), 488 (Argon) and 633 (HeNe) laser lines and an oil-immersion 63 × Zeiss Plan-Apochromat (1.4 NA, working distance 0.14 mm) objective. The following parameters were used: 1 × 1 binning, laser power 6%, gain 850, unidirectional line scanning, × 16 averaging, and 0.09 × 0.09 × 0.22 µm pixel size (XYZ). The microscope was controlled using the Zen Black (2.3) software, and images were acquired using the standard super-resolution mode (pinhole 2.77 AU). 3D Airyscan processing was done with Auto settings.

#### Stimulated emission depletion microscopy (STED)

An Abberior STED/RESOLF system (Abberior Instruments GmbH, Germany) equipped with a 100 × oil-immersion Olympus UPLSAPO objective (NA 1.4, working distance 0.13 mm) was used. Abberior-FIP-IgM was excited with a pulsed 635 nm laser and depleted using a 775 nm pulsed depletion laser, and emission signal was detected in the Cy5 channel (685/35 nm). Confocal images of Alexa Fluor® 488 anti-IgM F(ab)’_2_ were taken using the 488 nm laser, and the emission signal was detected in the GFP channel (525/25 nm). A 20 nm × 20 nm pixel size was used.

#### Huygens deconvolution

Images acquired with the SDCM (fixed samples) were deconvolved when indicated with Huygens Essential version 16.10 (Scientific Volume Imaging, The Netherlands, http://svi.nl), using the Quick MLE algorithm, Theoretical PSF mode and Auto Iteration Mode.

#### Super-resolution radial fluctuations (SRRF)

For SRRF processing^23,24^, 100 frames (135 × 135 pixels) were used for the reconstruction. For live imaging, 50 × 20 ms frames (85 × 85 pixels) were used. The parameters were as follows: 2.3 Ring Radius, 9 Radiality Magnification and 7 Axes in Ring. All other parameters were run in default mode. Parameters were defined using SQUIRREL analyses (resolution-scaled error (RSE) and resolution-scaled Pearson (RSP) values)^25^.

#### Content-aware image restoration (CARE)

For 4D (xyzt) imaging, movies were recorded using a SDCM and a Photometrics Evolve camera as described before. After imaging, samples were fixed with 4% PFA, and pairs of low signal-to-noise ratio (20 ms exposure time, as used for 4D live imaging) and high signal-to-noise ratio (500 ms) images were acquired. These images were used for training as input and target images, respectively. The CARE 2D model was trained from scratch for 100 epochs on 700 paired image patches (image dimensions: (100,100), patch size: (16,16)) with a batch size of 16 and a Laplace loss function, using the CARE 2D ZeroCostDL4Mic notebook (v1.12)^26,27^. Key python packages used include TensorFlow (v1.15), Keras (v2.3.1), CSBDeep (v0.6.2), NumPy (v1.19.5), CUDA (v11). The training was accelerated using a Tesla T4 GPU. Default Advanced Parameters were enabled, and no augmentation was used for training. The model was validated using unseen images, obtaining a structural similarity index measure (mSSIM) of 0.723.

#### Image analysis

Deconvolved images (fixed samples, SDCM) were used for colocalisation analysis. Colocalisation analyses based on Mander’s overlap coefficient^28^ were performed in Fiji^29^ using the Colocalisation Threshold tool. As negative control to assess random colocalisation, one of the channels was rotated 90° left, and Mander’s coefficients were measured again. Mean fluorescence intensity (MFI) was measured from the raw images (sum intensity projection; 100 × 100 circular ROI) using ImageJ. MFI of the background was measured in 4 random positions per image and subtracted from the MFI of the cells. Particle tracking was done using Trackmate in Fiji^30^. To compare the resolution of the different techniques, the full-width-at-half-maximum (FWHM) value was used to determine the diameter of the vesicles. The line profile tool was used in ImageJ^31^ and the fluorescence intensity profiles were fitted to a Gaussian curve using Curve Fitter in Fiji or Solver in Excel. The FWHM of this curve was calculated for each profile to obtain the final measurements (5–10 vesicles per condition).

### Statistical analysis and illustrations

The mean values of each experiment (n = 3) were used to calculate statistical significances in GraphPad Prism 6 using a paired Student’s *t*-test. Statistical values are denoted as: *P < 0.05, **P < 0.01, ***P < 0.001, ****P < 0.0001. Graphs were created in GraphPad Prism 6 or SuperPlotsOfData^32,33^ and illustrations were created with BioRender. Figure formatting was done in Inkscape version 1.0.0.

## Results

### FIP-IgM does not perturb the normal internalisation and traffic of BCR

**Figure 1:**
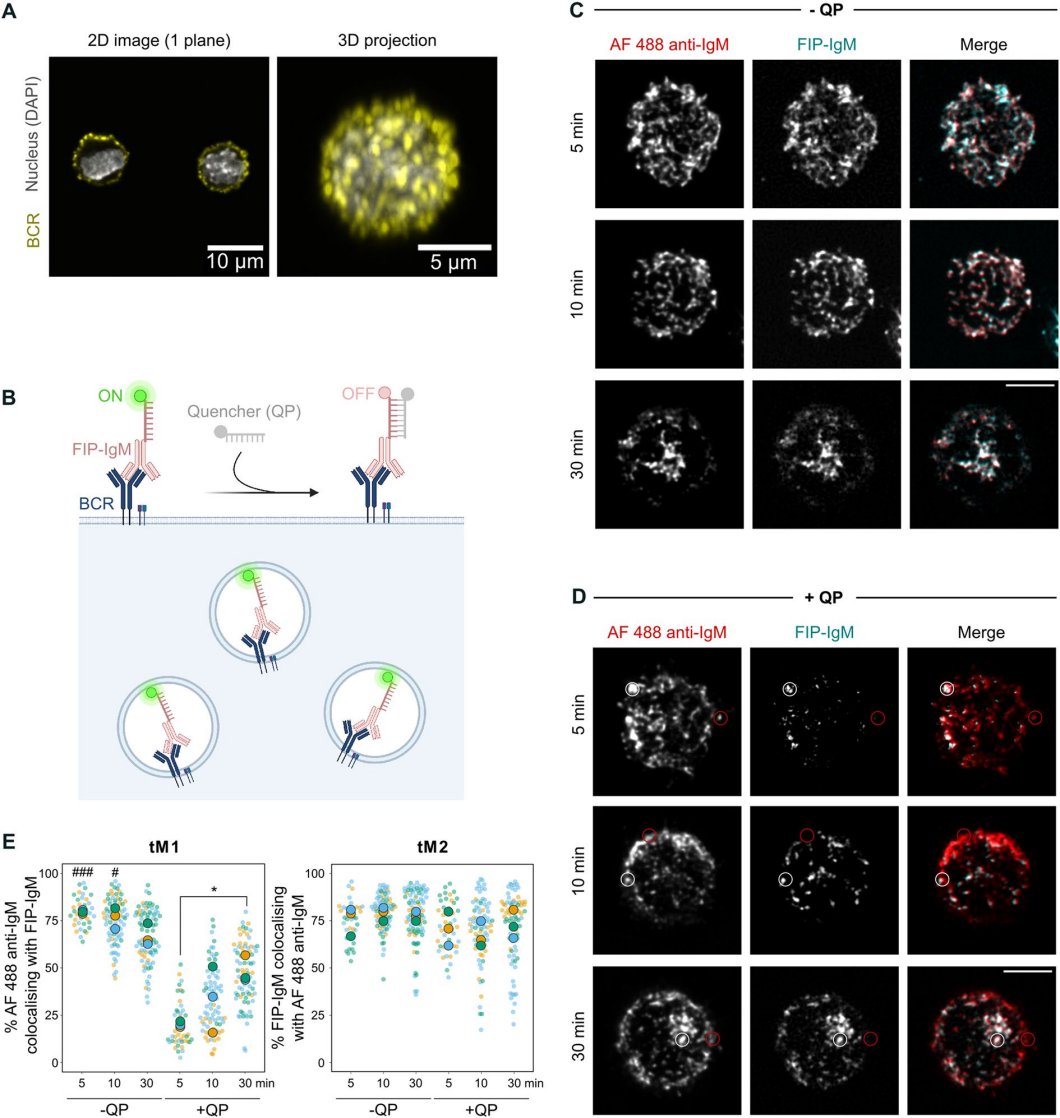
The SHIP assay can be used to track antigen internalization in B cells. (**A**) A20 D1.3 B cells imaged with a SDCM. Cells were stained on ice with AF488 anti-IgM to visualize the surface-bound BCR, fixed and stained with DAPI (nucleus). (**B**) Schematic of the SHIP assay. B cells are incubated with FIP-IgM as a surrogate antigen to label and activate the BCRs. B cell activation will trigger antigen/BCR internalization. After QP addition, the surface-bound probes are quenched and only the signal from the internalized antigen will be detected. (**C**,**D**) B cells were stained with ATTO-FIP-IgM and anti-IgM Alexa Fluor 488, and activated at different time points. After activation, cells were incubated with (**C**) PBS or (**D**) QP for 10 min and fixed. Images were acquired using a SDCM. A representative image of one cell (stack, sum intensity projection) is shown. White and red circles mark examples of internalised and non-internalised antigen, respectively. Scale bar: 5 µm. (**E**) Colocalisation analysis of the images shown in C-D. Images were deconvoluted with Huygens and colocalisation was measured using Mander’s overlap coefficients (tM1 = % of the anti-IgM Alexa Fluor 488 signal colocalising with ATTO-FIP-IgM signal; tM2 = % of the ATTO-FIP-IgM signal colocalising with anti-IgM Alexa Fluor 488 signal). Different colours represent independent experiments (n = 3): the mean of each experiment is shown with large symbols, and small symbols show the measured cells. Statistics were calculated using the mean of each experiment (n = 3) and a paired t-test (*P < 0.05; ^#^*P* < 0.05 vs 10 min + QP; ^###^*P* < 0.001 vs 5 min + QP).

Intracellular vesicular pathways have been primarily studied in cultivated fibroblasts or cancer cells that adhere firmly to a substrate and form a large spread area covering 600–1600 µm^2^^34^, depending on the cell type and conditions. However, the detection of intracellular vesicles becomes particularly challenging in small (< 100 µm^2^) or round cells. When studying the internalisation of membrane receptors or extracellular cargo, precise detection is further hindered by the remaining membrane-bound signal. Lymphocytes are round, non-adherent cells with a small cytoplasmic space, as the nucleus occupies 70% of the cell volume, and a high receptor concentration on their surface (Fig. 1A)^7–9,35^. With the aim to find a solution for these obstacles, we set up the SHIP assay to image internalized antigen in B cells. The SHIP assay requires two different oligos: a fluorescent ssDNA internalisation probe, that is conjugated to the desired ligand or antibody (FIP; 5′ fluorophore-TCAGTTCAGGACCCTCGGCT-N3 3′), and a complementary ssDNA-containing quenching probe (QP; 5′ AGCCGAGGGTCCTGAACTGA-Quencher 3′) (Fig. 1B). Previous studies have shown efficient labelling of antibodies and nanoparticles with Cy5-FIP and quenching using a Black Hole Quencher (BHQ) 2-labelled quenching probe (QP-BHQ2).

Here, based on our aim to evaluate the power of the SHIP assay for microscopy, we chose Abberior® STAR 635P-FIP (Abberior-FIP) and ATTO 647N-FIP (ATTO-FIP) as FIP fluorophores. These fluorophores have photophysical properties superior to those of Cy5, including better brightness and photostability, and they are also well suited for super-resolution microscopy. As quencher, the dark quencher BHQ3 (λ 620–730; λ_max_ 672) was selected over BHQ2 (λ 550–650; λ_max_ 579) due to its better spectral overlap with Abberior® STAR 635P (λ_max_ 651) and ATTO 647N (λ_max_ 664). As our model system, we chose A20 D1.3 B cells expressing an IgM BCR. Anti-IgM antibodies, commonly employed as surrogate antigens to activate B cells of any specificity, were labelled with Abberior-FIP and ATTO-FIP and used to engage the BCRs and trigger receptor-mediated endocytosis and antigen processing. A good degree of labelling was achieved with both probes, as cells stained with the FIP-labelled anti-IgM antibodies (FIP-IgM) displayed a similar intensity shift as those labelled with commercial Alexa Fluor® (AF) 647 anti-mouse IgM (Fig. S1A).

Next, we investigated whether the SHIP assay could be used to study antigen traffic in B cells without interfering with normal BCR internalisation. Using flow cytometry, we analysed the IgM internalisation rate in cells activated with Abberior- or ATTO-FIP-IgM at different time points. As a control, cells activated with a commercially available biotinylated anti-IgM were used. We found no differences in BCR internalization induced by the FIP-IgM probes or the biotinylated anti-IgM (Fig. S1B–D). These results were consistent with the literature, as the Cy5-FIP (7 kDa) has been described not to interfere with large molecules such as antibodies^36^. We observed, though, a slight yet notable decrease in the intensity of the total FIP-IgM signal over time in the absence of QP (Fig. S1C–D; red histograms). We hypothesized that this decrease could be due to (1) sensitivity of the fluorophores to the low pH endosomal environment, (2) antibody-mediated receptor shedding, or (3) fast antibody off-rates. To test this, we compared the FIP-IgM antibodies to other commercial and in-house labelled anti-IgM antibodies over time. B cells were incubated with ATTO-FIP-IgM, Abberior-FIP-IgM, and the same polyclonal anti-IgM antibody batch conjugated in-house to ATTO 647, allowing direct comparison of the antibody binding. Additionally, commercial anti-IgM antibodies labelled with AF 633 (pH-insensitive) and FITC (pH-sensitive) were used as controls for pH sensitivity. The signal of the AF 633 and ATTO 647 antibodies remained unchanged over time, as expected for pH-insensitive dyes, but also demonstrating that the used anti-IgM antibodies, labelled in-house or commercially, did not induce receptor shedding and bound stably to the target (Fig. S1E). The results implied that the IgM-FIP probes possessed some intrinsic level of instability in B cells which could be linked to the acidity of the antigen processing endosomal compartments or other specific features of our model system. However, this finding was not expected to pose major problems for imaging applications. In addition, we also experimentally verified that the addition of the QP did not affect cell viability, unlike the acid wash treatment classically used to strip cell surface-bound molecules (Fig. S1F) and that the FIP-IgM probes, but not the FIP ssDNA oligo alone, triggered normal B cell activation, using CD19 and Syk phosphorylation as readout (Fig. S1G).

Next, to evaluate the performance of the SHIP assay in microscopy, we engaged the BCRs with ATTO-FIP-IgM, Abberior-FIP-IgM, or AF 488-labelled anti-IgM as control. B cells were activated for different time points (5, 10, and 30 min), followed or not by quenching with the QP. Samples were fixed and imaged with a spinning disk confocal microscope, and images were deconvolved (Fig. S2A). As expected, an extremely high degree of colocalisation, compatible with non-disturbed BCR internalisation and correct endosomal targeting, was found between the FIP-IgM and the AF 488 anti-IgM antibodies before treatment with the QP (Fig. 1C,E; Abberior-FIP-IgM *data not shown*). After the addition of the quenching probe, a dramatic decrease in the FIP-IgM intensity was detected, especially at early time points, consistent with the flow cytometry analysis (Fig. S2B–D). Since only a small percentage of the antigen was internalised at 5 min (< 10%; Fig. S1B), few vesicles were visible in the quenched samples at that time (Fig. 1D). The number of antigen vesicles increased over time, and after 30 min most of the FIP signal remained detectable after the addition of the quencher, in accordance with the majority of the antigen (around 60%; Fig. S1B) being internalised at that point (Fig. 1D,E). As in the flow cytometry experiments, we also observed a decay in the total signal at later time points before the addition of the quencher (Fig. S2C). Nevertheless, the weakening of the signal did not seem to interfere with the imaging, and we often even noticed brighter intracellular signal using FIP-IgM compared to AF 488 anti-IgM, especially notable later in live imaging (see Fig. 3B or Supplementary Movie 2).

### The SHIP assay improves the analysis of internalised antigen at early time points

**Figure 2:**
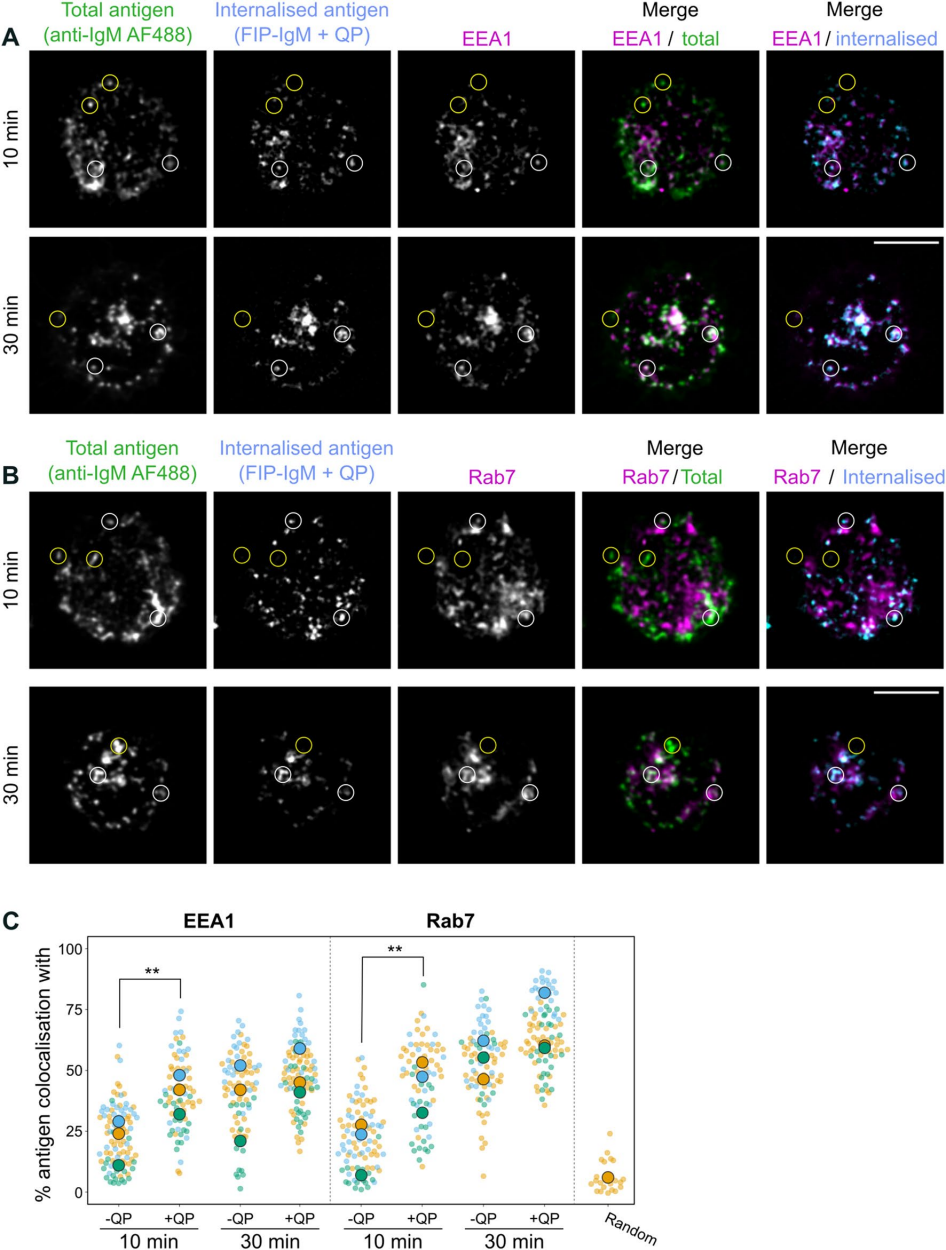
Colocalisation of the antigen with early and late endosomal markers. (**A**,**B**) B cells were engaged with ATTO-FIP-IgM and anti-IgM AF488, and activated for 10 or 30 min at 37 °C. After activation, cells were incubated with QP for 10 min on ice, fixed and stained for (**A**) Rab7 or (**B**) EEA1. Images were acquired using a SDCM. A representative image of one cell (stack, sum projection) is shown. The white and yellow circles mark an example of internalised or non-internalised antigen, respectively. Scale bar: 5 µm. (**C**) Colocalisation analysis of the internalised antigen with EEA1 and Rab7. B cells were stained with ATTO-FIP-IgM and activated for 10 or 30 min. After activation, cells were incubated with PBS (−QP) or QP (+QP), fixed and stained for EEA1 and Rab7. Images were deconvoluted with Huygens and colocalisation was measured using thresholded Mander’s overlap coefficient (tM1 = % of antigen colocalising with EEA1 or Rab7). Different colours represent independent experiments: the mean of each experiments is shown with large symbols, and small symbols show the measured cells. As a control for random colocalisation, one channel was rotated 90° and colocalisation was measured. **P < 0.01, paired t-test (n = 3).

In conventional immunofluorescence studies, it is impossible to unambiguously distinguish the internalised BCR-antigen complexes from the surface-bound receptors. Inaccurate determination of the signal can skew, for instance, the colocalisation analysis with vesicle markers, lowering the colocalisation coefficients and potentially leading to misguided biological interpretations. Thus, we proceeded to test the performance of the SHIP assay for antigen/endosome colocalization analysis. Typically, two different stages can be observed when studying antigen internalisation. At early timepoints (5–20 min) vesicles appear disperse and at late timepoints (15–45 min) the vesicles start clustering in the nuclear groove. Here, we selected two different activation times, one for early activation (10 min) and one for late activation (30 min), and prepared immunofluorescence samples using ATTO-FIP-IgM and AF 488 anti-IgM as a control. After this time, samples were quenched, fixed, and stained for the late endosome (LE) marker Rab7 or the early endosome (EE) marker EEA1 to compare the colocalisation of the total antigen (AF 488 anti-IgM) and the internalised antigen (ATTO-FIP-IgM) with the vesicle markers. As observed in the images, internalised vesicles (FIP-IgM^+^/AF 488^+^, white circles) were commonly found together with Rab7 or EEA1, unlike the non-internalised antigen (FIP-IgM^−^/AF 488^+^, yellow circles) (Fig. 2A,B). Moreover, in the later time point, the internalised FIP-IgM was found to colocalise with Rab7^+^ endosomes close to the perinuclear compartment, as previously reported^7,37,38^, indicating once more that the FIP-IgM probe did not perturb normal antigen trafficking.

In order to measure the improvement provided by the SHIP assay, we compared the colocalization between the antigen (ATTO-FIP-IgM) and the vesicle markers in samples treated or not with the QP (Fig. 2C; Fig. S3). The conditions without QP resemble the conventional setup where the extracellular and internalized ligands are not distinguished. Colocalisation of antigen with EEA1 and Rab7, measured by Mander’s overlap coefficients, showed a significant increase, from 25 to 45%, at early time points (10 min) when using the SHIP assay (+QP; Fig. 2C). On the other hand, when the quencher was added at later time points (30 min), no significant differences were observed in the colocalisation, as the majority of the antigen was already internalised at that time, and most of the vesicles had moved towards the perinuclear area. These results demonstrate that the SHIP system can provide a valuable benefit to the colocalisation analysis of cellular cargo, especially shortly after internalisation.

### The SHIP assay allows for specific imaging of internalised antigen in living cells

**Figure 3:**
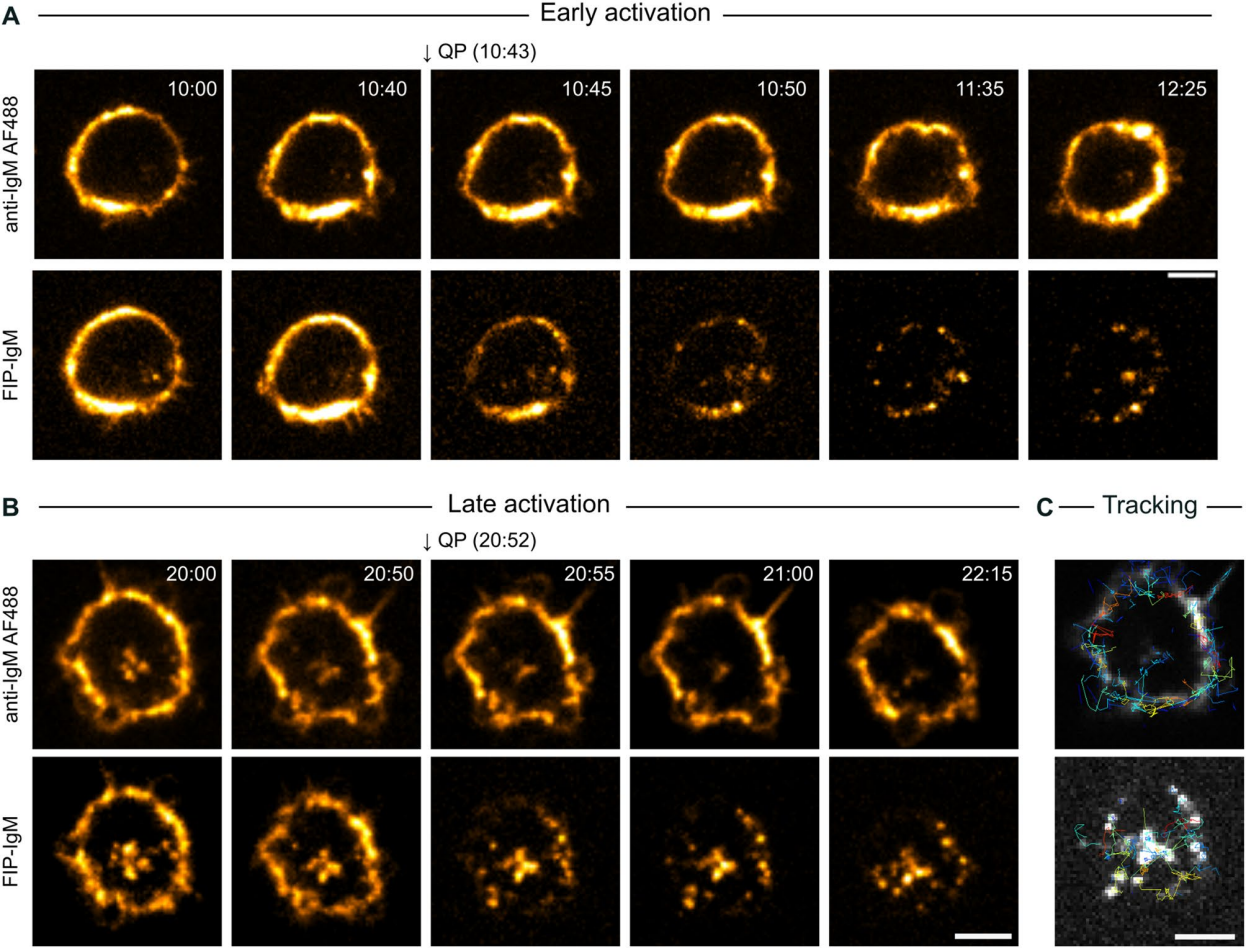
The SHIP assay improves live cell imaging of antigen trafficking. (A-B) B cells were attached to a fibronectin-coated dish and engaged on ice with ATTO-FIP-IgM and Alexa Fluor 488 anti-IgM. Cells were transferred to 37 °C for (**A**) 10 min or (**B**) 20 min, for antigen/receptor endocytosis. The recording was started and after 40–60 s QP was added to the cells without interrupting image acquisition. QP addition is indicated with an arrow. Images were acquired using a SDCM (one slice, one frame every 5 s). Time in minutes after triggering endocytosis is indicated in the top-left corner. LUT (Look Up Table) Hot Yellow. (**C**) Antigen tracking after quenching was performed using TrackMate. The antigen tracks (blue for shorter and red for longer) are shown in the image. Scale bar: 5 µm.

To date, there are no reports showing the use of the SHIP assay in living cells. Thus, we next evaluated the potential of the SHIP assay for live imaging. B cells were attached to a fibronectin-coated microscopy dish and engaged with ATTO-FIP-IgM or AF 488 anti-IgM as a control. The time points after receptor triggering/FIP addition when the quencher was added were defined as early (< 15 min; Fig. 3A and Supplementary Movie 1) or late (> 15 min; Fig. 3B and Supplementary Movie 2). Images were recorded for at least 30 s before the addition of the quencher without stopping the acquisition. Only a few seconds after QP addition, the extracellular FIP signal was dramatically quenched, while the control AF 488 signal remained unmodified. Notably, after quenching the non-internalised signal, the internalised vesicles became more visible and distinct, improving vesicle tracking (Fig. 3C and Supplementary Movie 3).

**Figure 4:**
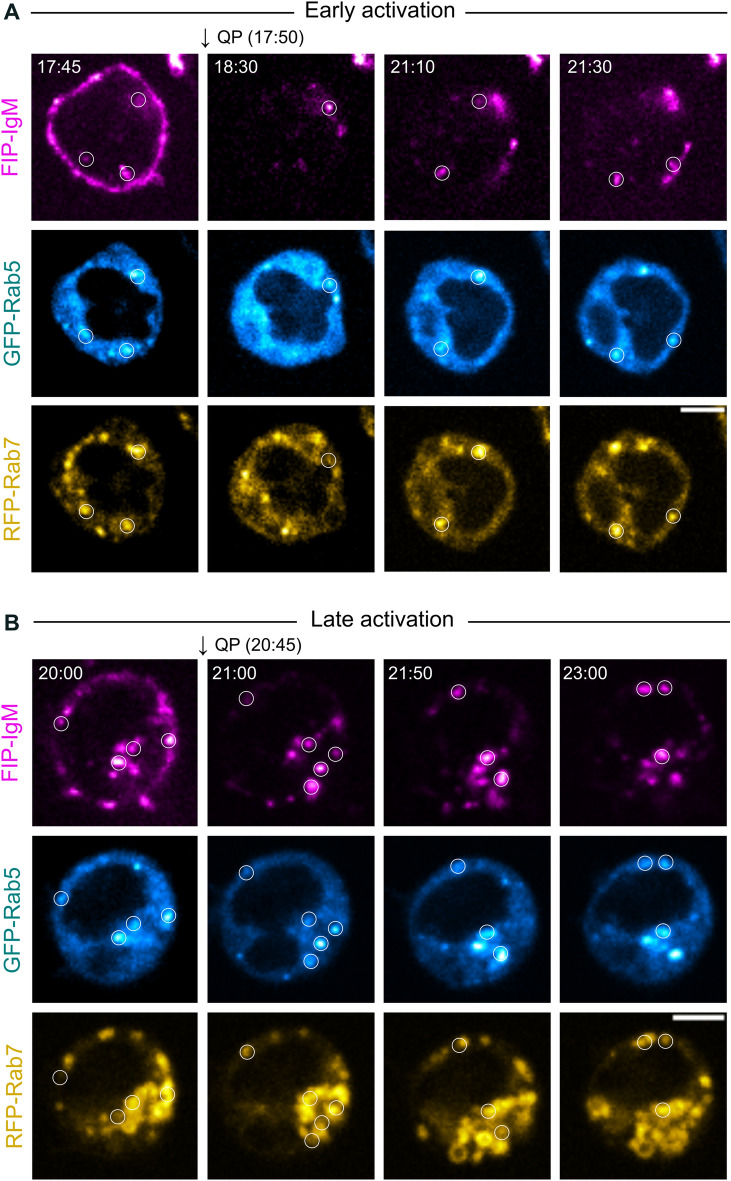
Internalised antigen colocalizes with endosomal markers in live imaging. (**A**,**B**) B cells were transfected with GFP-Rab5 and RFP-Rab7 and engaged with ATTO-FIP-IgM on ice. Cells were transferred to 37 °C to trigger endocytosis and the recording was started. QP was added to the cells at indicated time points (arrows) after activation without interrupting image acquisition. Images show an early (**A**) or late stage (**B**) of antigen traffic. Images were acquired using a SDCM (one slice, one frame every 5 s). Time in minutes after triggering endocytosis is indicated in the top-left corner. Scale bar: 5 µm. LUTs: Hot Magenta, Hot Cyan and Hot Yellow.

In our previous work, we found a considerable overlap of the internalised antigen with both early and late endosomal markers, as well as with Lysotracker, a fluorescent dye that stains acidic compartments, suggesting that antigen is directed to specialised processing vesicles immediately after internalization^7^. However, challenged by the surface-derived antigen signal, we were able to clearly distinguish a few vesicle internalization events because of the overlapping surface-derived signal. Now, to tackle this issue, we utilized the SHIP to follow the internalized vesicles together with endosomal markers or LysoTracker. Consistently with our previous report, when activating the cells with ATTO-FIP-IgM, we found that some antigen vesicles colocalised with GFP-Rab5 (EE marker) and RFP-Rab7 (LE marker) (Fig. 4A,B; Fig. S4A; Supplementary Movie 4A-B and 5), and LysoTracker (Fig. S4B and Supplementary Movie 6). After QP addition, the vesicles were clearly distinguishable and showed colocalisation with the vesicle markers, demonstrating that the internalised antigen was indeed often moving close to or together with acidic vesicles positive for early or late endosomal markers.

**Figure 5:**
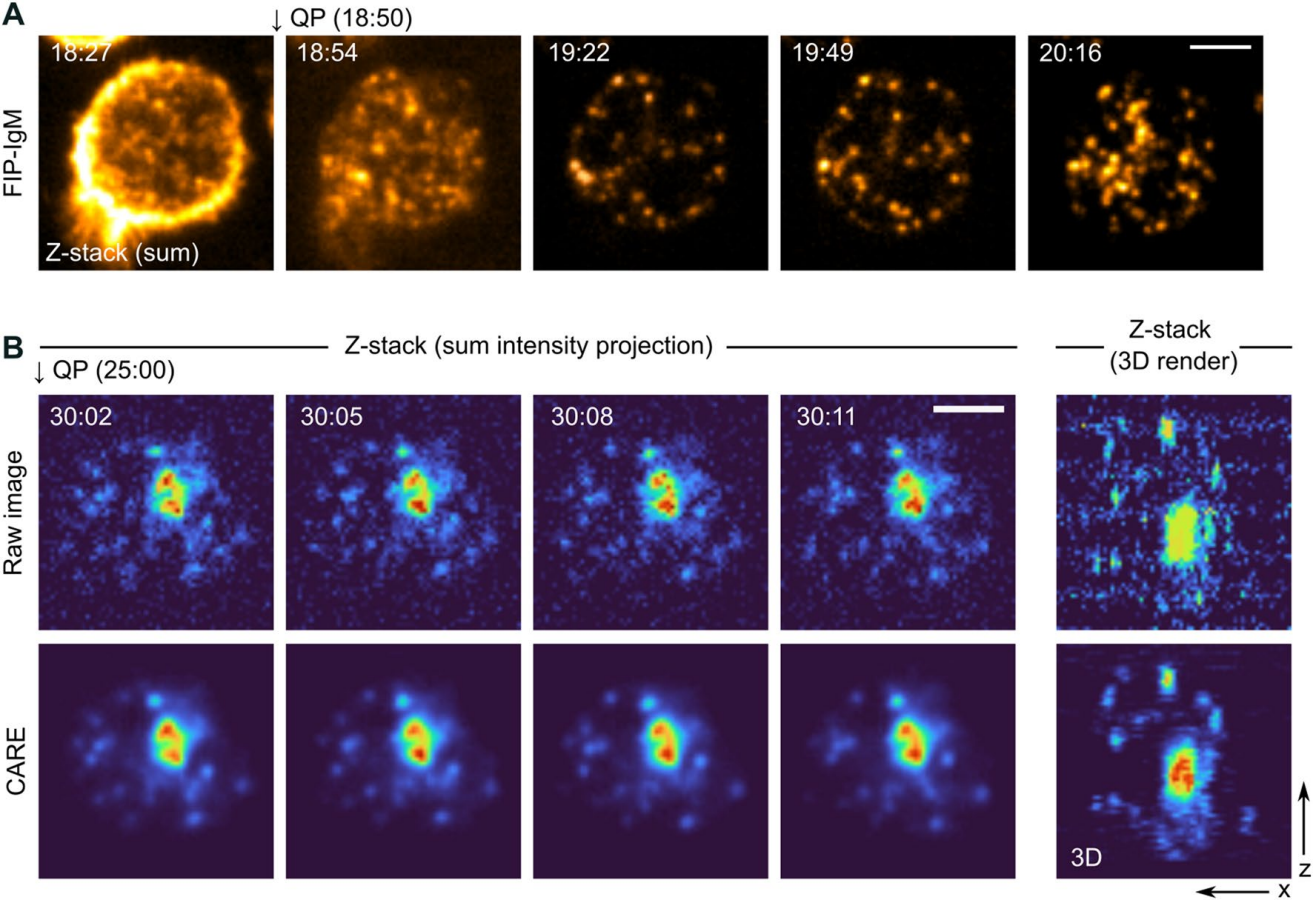
4D live imaging of antigen in B cells. (**A**) B cells adhering to fibronectin were engaged with ATTO-FIP-IgM on ice. Cells were transferred to 37 °C to trigger endocytosis and the recording was started. QP was added to the cells at indicated time points (arrows) after activation without interrupting image acquisition. Images were acquired using a SDCM (49 planes, one stack every 27 s). Scale bar: 5 µm. LUT Hot Yellow. (**B**) Short acquisition time images (raw image) were acquired using a SDCM (49 planes, one stack every 3.7 s) and restored using CARE. Time in minutes after triggering endocytosis is indicated in the top-left corner. Scale bar: 5 µm. LUT Turbo.

Next, we assessed the capabilities of the SHIP assay in 3D live imaging (i.e. 4D: XYZT). B cells were engaged with ATTO-FIP-IgM as above and imaged before and after the addition of the quencher. An exposure time of 500 ms was used, and every time point was composed of 49 slices, allowing an average imaging speed of 27 s per volume. After quenching the surface signal, the separation of the internalised vesicles from the plasma membrane became drastically better (Fig. 5A; stack image, sum intensity projection). In order to push the speed limits of image acquisition, we turned to deep learning-based image restoration. Now, an exposure time of 20 ms was used, allowing an average imaging speed of 3–4 s per volume (49 slices). Images were then restored using content-aware image restoration (CARE^27^) to obtain the final 3D movie (Fig. 5B and Supplementary Movie 7). The results showed that the ATTO-FIP-IgM signal was sufficiently stable over time and bright enough to perform 4D live imaging for at least up to 20 min. Longer acquisition times were not tested. In this way, we could image and analyse a considerably higher number of antigen vesicles than earlier possible.

### The SHIP assay is compatible with super-resolution imaging

**Figure 6:**
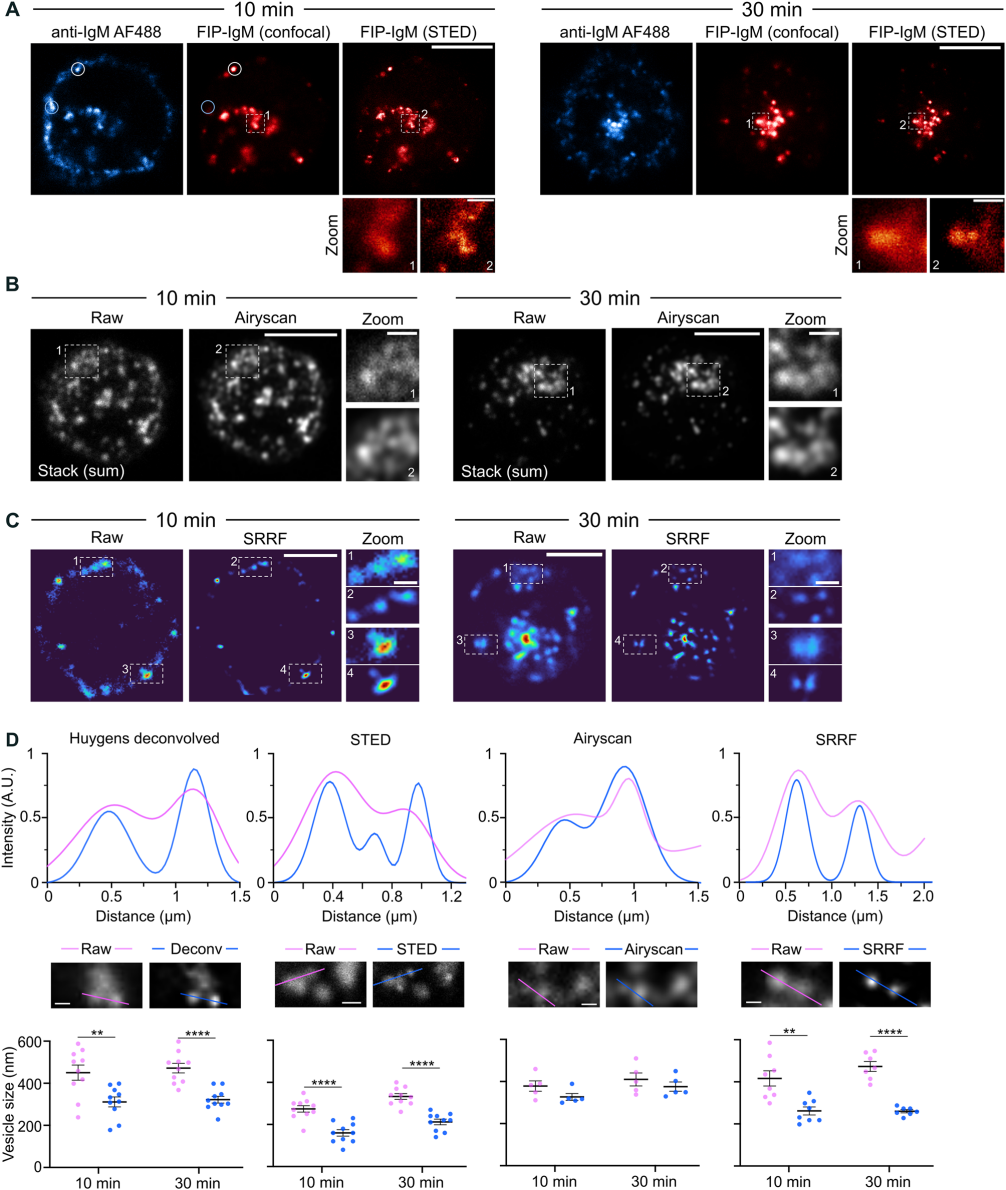
Super-resolution imaging of internalised BCR-antigen complexes using the SHIP assay (**A**) B cells were activated with Abberior-FIP-IgM and AF488 anti-IgM for 10 and 30 min, quenched with BHQ3-QP, fixed and imaged with Abberior STED. A representative image (1 slice) of each channel is shown. The white circle shows one example of an internalized vesicle, and the blue circle marks an example of non-internalised antigen. The dashed rectangle is shown as a zoom inset. Scale bar: 5 µm; scale bar of the inset: 500 nm. LUTs: Hot Cyan and NanoJ-Orange. (**B**) B cells were activated with ATTO-FIP-IgM for 10 and 30 min, quenched with BHQ3-QP, fixed and imaged with Airyscan. A representative stack image (sum intensity projection) is shown. The dashed rectangle is shown as a zoom inset. Scale bar: 5 µm; scale bar of the inset: 1 µm. (**C**) B cells were activated with ATTO-FIP-IgM for 10 and 30 min and imaged with SDCM. A total of 100 frames per image were acquired to perform SRRF. A representative image (once slice) is shown. Scale bar: 5 µm; scale bar of the inset: 1 µm. (**D**) Representative profile plots (upper row) comparing the apparent vesicle size of the different imaging techniques. The images (middle row) show the image regions and the line scan position used for the profile plots and the plots (bottom row) with the FWHM of the curves before (pink) and after (blue) super-resolution. **P < 0.01, ****P < 0.001; paired t-test (n = 5–10).

Due to the diffraction limit of light, traditional confocal microscopy cannot resolve structures below 200–250 nm. Since intracellular vesicles often fall close to or below this resolution limit, we tested whether the SHIP assay could be combined with super-resolution imaging techniques. First, stimulated emission depletion (STED) microscopy was tested^39^. B cells were labelled with Abberior-FIP-IgM and AF 488 anti-IgM F(ab)’_2_, activated for 10 and 30 min, quenched, fixed, and imaged using an Abberior STED system. Conventional scanning confocal images of both channels were acquired, and in addition, the Abberior-FIP-IgM channel was also acquired in STED mode (Fig. 6A). The apparent diameter of the vesicles was determined by measuring the full width at half maximum intensity (FWHM) of the Gaussian curve of the FIP-IgM fluorescence. Indeed, STED imaging considerably reduced the apparent vesicle size (from 280–330 to 165–210 nm). As STED requires a relatively strong fluorescence signal, detecting the FIP signal was, however, challenging after quenching at early time points when only a limited amount of antigen had been internalised (Fig. S2C–D). Next, we tested two other high-resolution imaging systems that are often more easily accessible to researchers: an Airyscan confocal microscope^40,41^ and super-resolution radial fluctuations (SRRF) on a spinning disk confocal microscope^24^. B cells were stained with ATTO-FIP-IgM, activated for 10 or 30 min, quenched, fixed, and imaged. An LSM880 microscope with Airyscan was used to image the cells in 3D (z-stack; Fig. 6B). For SRRF, 100 frames per image (1 slice) were acquired using a SDCM and then processed in Fiji (Fig. 6C). Airyscan images showed a modest decrease in the apparent vesicle size (from 380–400 to 330–370 nm), similar to those images acquired with a SDCM and deconvolved using Huygens (from 450 to 320 nm) (Fig. 6D, Fig. S2A). SRRF, on the other hand, showed a good improvement (from 450 to 260 nm), but this was limited to one optical section. Although Airyscan and SRRF are compatible with live-cell imaging, these methods require a bright signal to obtain high-quality data. Resulting from the small number of internalised vesicles, the low brightness of the samples at early time points hampered the acquisition of live super-resolution images (data not shown) in our model system. Nevertheless, we demonstrated that the SHIP assay is compatible with several super-resolution approaches using fixed samples. The resolution increase, especially in STED and SRRF, clearly improved the separation of the vesicles, also unveiling small vesicles located close to each other. Hence, the SHIP assay could be applied in future research to facilitate the study of internalised cargo and receptors in different cell types.

## Discussion

In the last two decades, microscopy has become an increasingly powerful tool to study dynamic cellular events, increasing our understanding of the complexity of intracellular vesicle traffic. However, distinguishing between internalised and non-internalised ligands, especially in small cells such as lymphocytes, remains a major challenge even with modern high-resolution techniques. A tool for analysing internalisation, termed Specific Hybridization Internalisation Probe (SHIP), uses a fluorescence internalisation probe (FIP) and a complementary quenching probe (QP) to unambiguously separate the internalised receptors or ligands from those on the plasma membrane^15^. While the assay has been used in a handful of studies to measure internalisation using flow cytometry, the potential of this system for advanced microscopy studies has remained entirely unexplored. Our study demonstrates the high potential of the SHIP assay for improving the imaging of receptor/ligand internalization and the associated vesicle compartments and shows that the SHIP assay can be adapted to multiple imaging modalities, including live imaging and super-resolution microscopy.

Here, we investigated the functionality of the SHIP system using B cells as a model system. We validated and employed ATTO/Abberior-FIP-labelled anti-IgM antibodies as a surrogate antigen to trigger BCR activation and antigen/BCR internalisation and trafficking (Fig. 1; Fig S1). As expected, the addition of the QP efficiently quenched the extracellular signal enabling crisp detection of the internalised receptors (Fig. S1C–D and S2B). When evaluating the advantages of the SHIP system for colocalisation studies in immunofluorescence samples, we detected a significant increase in the colocalisation coefficients when analysing the internalised IgM (FIP-IgM + QP) together with vesicular markers soon after internalization as compared to the conventional labelling (Fig. 2). Upon BCR engagement, fast vesicle sorting and trafficking events occur within minutes but the imaging of these internalised receptors is strongly hindered by the bright signal of the yet non-internalized receptors. A better understanding of these intricate vesicle trafficking events would answer critical open questions about the mechanisms enabling processing of the internalized antigen into the peptides for MHCII presentation and, ultimately, mounting the life-saving high-affinity antibody responses. Using SHIP, the dramatic improvement in the definition of internalized antigen/BCR enabled precise vesicle detection and tracking (Figs. 2 and 3). In the later time points, when most of the antigen was already internalized, the improvement offered by SHIP remained modest.

As live imaging is a critical tool to understand the vesicle dynamics and maturation events, we proceeded to investigate the performance of the SHIP system in live cells. A remarkable improvement was observed after adding the quencher at both early and late time points (Fig. 3), as eliminating the bright fluorescence signal from the plasma membrane unveiled numerous endosomes close to the plasma membrane. We also tested the system in cells co-transfected with GFP-Rab5 and RFP-Rab7 or loaded with LysoTracker to visualize antigen colocalisation with these vesicle markers (Fig. 4 and Fig. S4). Using SHIP, we were able to unambiguously detect the vesicles where antigen colocalized with endosomal markers even when they were located at the close vicinity of the plasma membrane.

Since vesicles are highly dynamic structures that travel in three dimensions, live imaging in 2D can provide only a limited amount of information. The widely available spinning disk confocal microscopes equipped with a sensitive camera, such as EMCCD, can be used for 3D live imaging of rapid cellular processes, as opposed to classical confocal microscopes^42^. Using a SDCM, 3D volumes of B cells were acquired with a temporal resolution of 27 s without compromising signal quality (Fig. 5A). This speed, however, does not guarantee the capture of fast endosomal vesicle movements. Thus, in an attempt to gain higher temporal and spatial resolution, we combined the SDCM acquisition with an image restoration approach. In the last decade, machine learning and deep learning approaches have become popular tools for image analysis and image restoration. Here, content-aware image restoration (CARE), a deep learning-based image restoration algorithm^27^, was used to denoise 4D data sets. To train this network, pairs of images with a low and a high signal-to-noise ratio (SNR) were acquired after the fixation of the structures with PFA. We were able to improve the temporal resolution to 3.7 s per volume with this approach (Fig. 5B), which is comparable to the timeframes of 2.5–4.4 s achieved using lattice light-sheet microscopy, the fastest commercially available modality for 3D live imaging, to study endosomal trafficking in T lymphocytes^43–45^.

Super-resolution imaging has become increasingly popular in cell biology in the last two decades^46^. Super-resolution techniques include stimulated emission depletion (STED), single-molecule localisation microscopy (e.g. PALM and (d)STORM), structure illumination microscopy (SIM), expansion microscopy, pixel reassignment super-resolution techniques such as Zeiss Airyscan, and fluctuation-based super-resolution techniques, such as SRRF. These techniques have different strengths and weaknesses that need to be considered depending on the sample type, application, desired resolution, and, more importantly, availability to the researchers. We first tested the SHIP system using STED (Fig. 6A), as a theoretical resolution of 20–60 nm can be achieved with this system. The average size of the antigen-containing endosomes in B cells, measured in electron microscopy (EM) images, is around 100–200 nm for unilamellar vesicles and 200–400 nm for multilamellar vesicles^7^. Using SHIP in conjunction with STED imaging, we achieved an apparent vesicle size of 160 nm in good agreement with the previous EM data. Although traditional super-resolution modalities such as STED and single-molecule localisation microscopy provide the best resolution, these techniques require special microscopes and knowledge, and they are not yet widely applicable to 3D and live imaging. Hence, we next tested Airyscan, a Zeiss confocal microscope system well-suited for live imaging that has gained notable popularity in recent years, and SRRF, an algorithm compatible with most microscopes without hardware modifications. While Airyscan provided only a modest improvement in our setup (Fig. 6B,D), it allowed 3D imaging and improved the signal-to-noise ratio. SRRF, on the other hand, notably improved the imaging, but this algorithm can only be applied in 2D (Fig. 6C,D). Although both SRFF and Airyscan are compatible with live imaging, a bright signal is required to obtain high-quality images. In our study, following the published protocols^16^, we used a 2-fold molar excess of FIP to label the antibodies. Although a good brightness level was achieved, as measured by flow cytometry and microscopy (Fig. S1A and Fig. S2B–C), the limited amount of internalised antigen at early time points also limited the FIP-IgM signal remaining in the cells after quenching (Fig. 1B). As a result, we did not achieve good super-resolution live imaging data (data not shown), indicating that a higher fluorophore:antibody ratio should be used for such challenging live imaging super-resolution approaches. According to the literature, an optimal fluorophore:antibody ratio can be found between 2 and 8^47^, and therefore, it would be interesting to test in the future if a higher degree-of-labelling would further improve the imaging.

At the moment, the SHIP assay has only been used to track the internalisation of one ligand at a time. Therefore, in the future, it will be interesting to investigate the possibility to image two different ligands simultaneously using two pairs of complementary probes, such as ATTO647N-FIP/QP-BHQ3 and ATTO488-FIP/QP-BHQ1. A disadvantage of the SHIP system is, however, the high cost associated with the synthesis of the labelled oligos ($700–900 for every FIP and $200–300 for the QP). In addition, the FIP labelling of each ligand/antibody needs to be optimised. In order to omit the labelling step and utilize the same FIP for detection of different subjects, fluorescent FIP oligos could also be conjugated with biotin and attached to different streptavidin-labelled ligands or antibodies^18^.

In conclusion, this study explored for the first time the capabilities of the SHIP system for microscopy. SHIP can significantly improve the analysis of the internalized receptor/ligand, especially at locations close to the plasma membrane soon after internalization when the accurate detection of the vesicles is hampered by the membrane signal. We also found SHIP fully applicable for live and super-resolution imaging of internalised ligands and receptors. Together with its already described potential for high-throughput internalisation and recycling assays using flow cytometry, these properties confer the SHIP system broad applicability in different research fields, such as cell biology, immunology, and cancer biology.

## Supplementary Information

Supplementary Figures and Legends.

Supplementary Video 1.

Supplementary Video 2.

Supplementary Video 3.

Supplementary Video 4.

Supplementary Video 5.

Supplementary Video 6.

Supplementary Video 7.

Supplementary Video 8.
