# Supplementary material for: A specific hybridisation internalisation probe (SHIP) enables precise live-cell and super-resolution imaging of internalized cargo
Source: Sci Rep. 2022 Jan 12;12:620. doi: 10.1038/s41598-021-04544-6 (PMC8755761; doi:10.1038/s41598-021-04544-6)
Supplement: Supplementary file 1 — Supplementary Figures and Legends. [file 41598_2021_4544_MOESM1_ESM.docx]

**SUPPLEMENTARY INFORMATION**

**A Specific Hybridisation Internalisation Probe (SHIP) enables precise live-cell and super-resolution imaging of internalized cargo**

Running title: Enhanced imaging of internalized antigen using SHIP.

Sara Hernández-Pérez^1,2,3 *^ and Pieta K. Mattila^1,2,3 *^

^1^ Institute of Biomedicine and MediCity Research Laboratories, University of Turku, Finland

^2^ Turku Bioscience, University of Turku and Åbo Akademi University, Turku, Finland

^3^ InFLAMES Research Flagship Center, University of Turku

*** Corresponding authors:**

**Pieta Mattila**

E-mail: [pieta.mattila@utu.fi](mailto:pieta.mattila@utu.fi)

**Sara Hernández-Pérez**

E-mail: [sara.hernandezperez@utu.fi](mailto:sara.hernandezperez@utu.fi)

**Keywords:** Adaptive immune system; B cells; antigen trafficking; endosomes; internalisation; SHIP; super-resolution microscopy; live imaging; deep learning

**
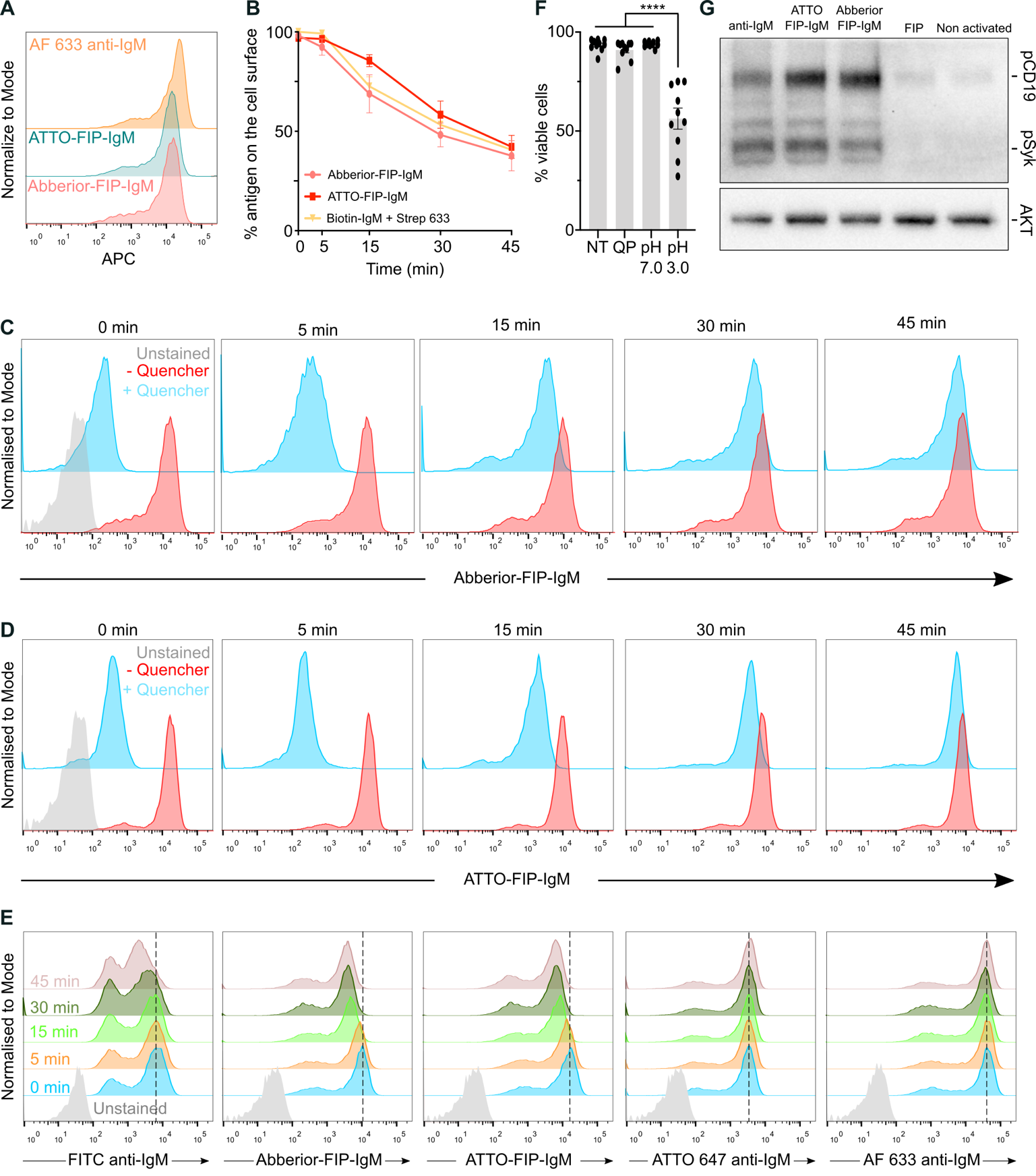
**

**Supplementary Figure 1. (A)** B cells were engaged on ice with a commercial AF633 anti-IgM antibody, ATTO-FIP-IgM or Abberior-FIP-IgM and analysed by flow cytometry. (**B-D)** BCR internalisation assay by flow cytometry. B cells were engaged on ice with Abberior-FIP-IgM (Abberior-FIP), ATTO 647N-FIP-IgM (ATTO-FIP) or biotinylated anti-IgM (Biotin-Strep). After the indicated time points, cells were placed on ice and quenched (FIP samples) or stained with Streptavidin 633 (Biotin-Strep samples) to analyse the amount of BCR inside or outside the cell respectively (n = 3; mean ± SEM). A representative experiment is shown in C (Abberior-FIP-IgM) and D (ATTO-FIP-IgM). **(E)** B cells were engaged on ice with a commercial FITC anti-IgM antibody, Abberior-FIP-IgM, ATTO-FIP-IgM, ATTO 647 anti-IgM or a commercial Alexa Fluor (AF) 633 anti-IgM antibody. Samples were then incubated at 37°C at different time points and fluorescence was analysed by flow cytometry. The dashed line indicated the fluorescence peak at 0 min. **(F)** B cells were incubated for 1 min in PBS (non treated; NT), quenching probe (QP), control buffer (pH 7.0) or acid wash buffer (3.0) and stained to assess the viability. The percentage of viable cells was determined using flow cytometry. N = 10; unpaired t-test, P-value **** < 0.001. **(G)** B cells were incubated with a commercial goat anti-mouse IgM, ATTO-FIP-IgM, Abberior-FIP-IgM or FIP alone (unconjugated oligo) for 15 min and subjected for Western blotting. As a control, non-activated cells (PBS) were used. The membrane was probed with anti-phospho CD19 and anti-phospho Syk antibodies to evaluate B cell activation. Total AKT was used as a loading control.

**
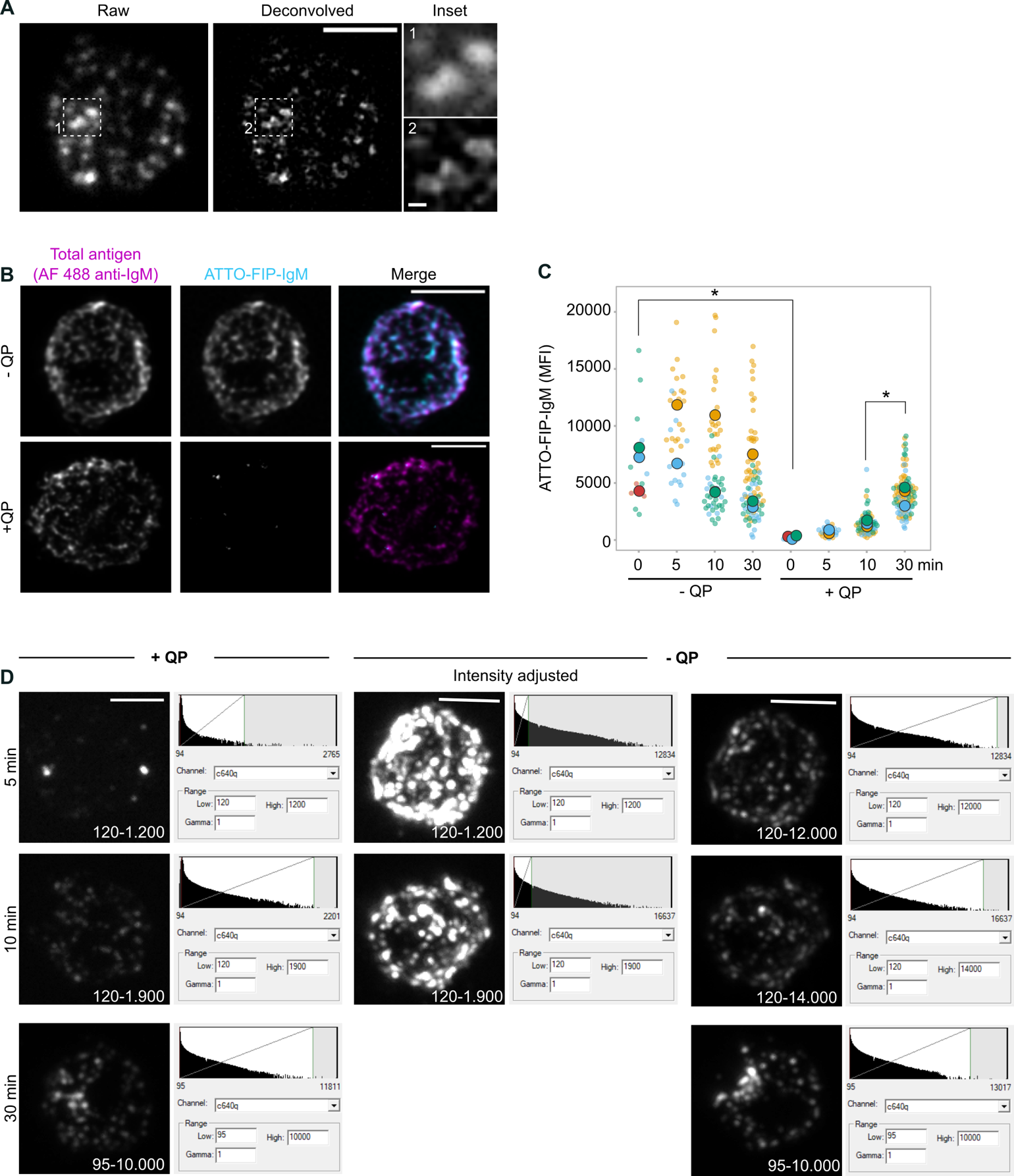
**

**Supplementary Figure 2 (A)** An example SDCM image of a cell activated with ATTO-FIP-IgM (10 min activation, quenched, fixed) before and after image deconvolution. The dashed rectangles are shown in the inset. Scale bar: 5 µm. Scale bar inset: 500 nm. **(B)** B cells were let to adhere on fibronectin-coated slides on ice and stained with ATTO-FIP-IgM (time 0 min), with and without addition of the quencher. Scale bar: 5 µm. (C) Mean fluorescence intensity (MFI) of ATTO-FIP-IgM measured in raw images (Z-projection, sum intensity) at different activation points with (+ QP) and without (- QP) quenching. The large symbols represent the mean of individual experiments (n = 3), and the small circles show the measured cells. Paired t-test (P * < 0.05). **(D)** Examples images of the B cells activated with ATTO-FIP-IgM quantified in (C). An example image (z-stack, sum intensity projection) is shown together with the min-max intensity values (in white, right lower corner of the image) and the intensity histogram (on the right). Scale bar: 5 µm.

**
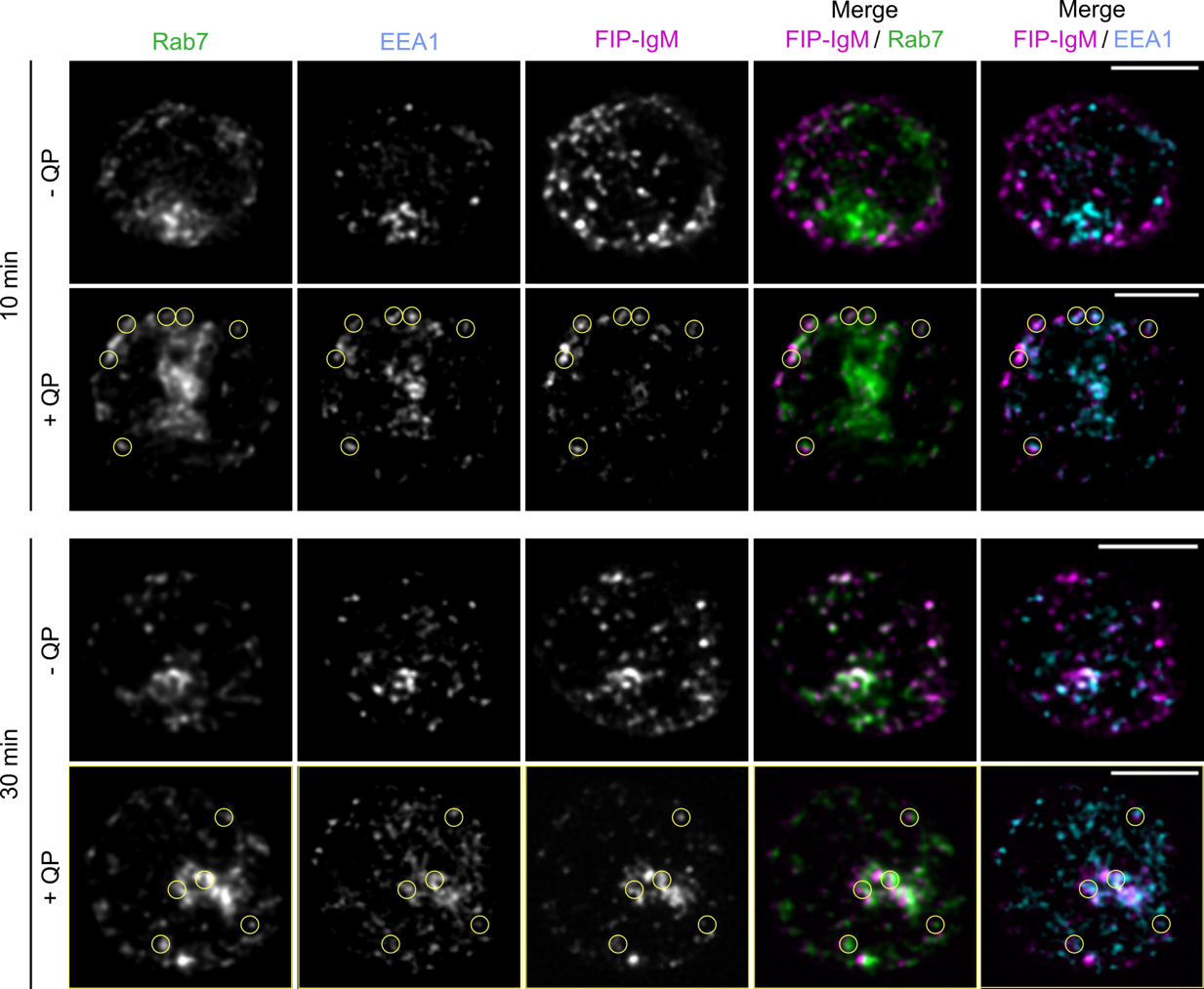
**

**Supplementary Figure 3, linked to Fig. 2C.** B cells were activated with ATTO-FIP-IgM for 10 or 30 minutes and treated or not with the QP. Cells were then fixed and stained for Rab7 (green) and EEA1 (cyan). An example image after deconvolution is shown. The yellow circles highlight examples of vesicles with antigen, Rab7 and EEA1. Scale bar: 5 µm.

**
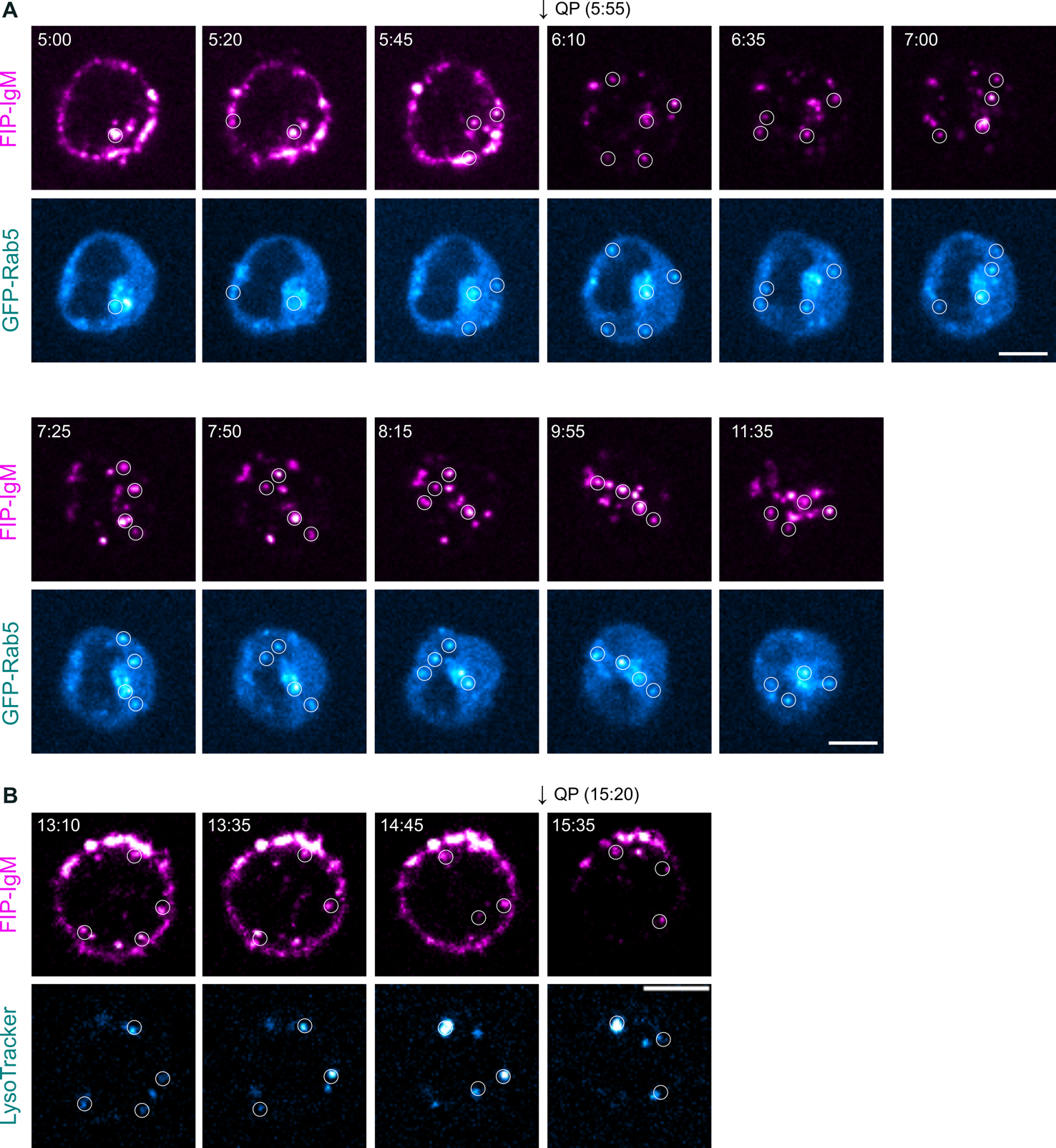
**

**Supplementary Figure 4.** B cells were **(A)** transfected with GFP-Rab5 or **(B)** loaded with LysoTracker Green and engaged with ATTO-FIP-IgM on ice. Cells were transferred to 37°C to trigger endocytosis and the recording was started. QP was added to the cells at indicated time points (arrows) after activation without interrupting image acquisition. Images were acquired using a SDCM (one slice, one frame every 5 s). Time in minutes after triggering endocytosis is indicated in the top-left corner. Scale bar: 5 µm. LUTs: Hot Magenta and Hot Cyan.

**Supplementary Movie 1.** B cells were attached to a fibronectin-coated dish and stained on ice with ATTO-FIP-IgM and AF 488 anti-IgM. Cells were transferred to 37°C for 10 min and recorded. After 40 s, QP was added to the cells without interrupting image acquisition. Images were acquired using a SDCM (one slice, one frame every 5 s). Left image, AF 488 anti-IgM; right image, ATTO-FIP-IgM. Scale bar: 5 µm. LUT Turbo.

**Supplementary Movie 2.** B cells were attached to a fibronectin-coated dish and stained on ice with ATTO-FIP-IgM and AF 488 anti-IgM. Cells were transferred to 37°C for 20 min and recorded. After 50 s, QP was added to the cells without interrupting image acquisition. Images were acquired using a SDCM (one slice, one frame every 5 s). Left image, AF 488 anti-IgM; right image, ATTO-FIP-IgM. Scale bar: 5 µm. LUT Turbo.

**Supplementary Movie 3.** Antigen tracking was performed on the cell shown in Supplementary Movie 2 after QP addition. Left image, AF 488 anti-IgM; right image, ATTO-FIP-IgM. The antigen tracks were colour-coded based on the length (blue for shorter tracks, and red for longer tracks).

**Supplementary Movie 4.** (A-B) B cells were transfected with GFP-Rab5 (cyan) and RFP-Rab7 (yellow), let to adhere on a fibronectin-coated dish and stained on ice with ATTO-FIP-IgM (magenta). Cells were transferred to 37°C for 15 min and recorded. QP was added to the cells without interrupting image acquisition. Images were acquired using a SDCM (one slice, one frame every 5 s). Scale bar: 5 µm. LUTs: Magenta Hot, Cyan Hot and Yellow Hot.

**Supplementary Movie 5.** B cells were transfected with GFP-Rab5 (cyan), let to adhere on a fibronectin-coated dish and stained on ice with ATTO-FIP-IgM (magenta). Cells were transferred to 37°C for 5 min and recorded. After 2:50 min (5:50), QP was added to the cells without interrupting image acquisition. Images were acquired using a SDCM (one slice, one frame every 5 s). Scale bar: 5 µm. LUTs: Magenta Hot and Cyan Hot.

**Supplementary Movie 6.** B cells were loaded with LysoTracker Green, let to adhere on a fibronectin-coated dish and stained on ice with ATTO-FIP-IgM (magenta). Cells were transferred to 37°C for 13 min and recorded. After 2 min (15:20), QP was added to the cells without interrupting image acquisition. Images were acquired using a SDCM (one slice, one frame every 5 s). Scale bar: 5 µm. LUTs: Magenta Hot and Cyan Hot.

**Supplementary Movie 7.** B cells were seeded on a fibronectin-coated dish and stained on ice with ATTO-FIP-IgM. Cells were transferred to 37°C. QP was added, and cells were recorded after that. Images were acquired using a SDCM. An exposure time of 20 ms was used, and 3D stacks were acquired (1 stack every 3.778 s). Movies of the raw data (left) and restored data (right) are shown. Upper panel: 3D reconstruction. Lower panel: sum intensity projection. Scale bar: 5 µm. LUT Turbo.
